# *Arabidopsis thaliana* myosin XIK is recruited to the Golgi through interaction with a MyoB receptor

**DOI:** 10.1038/s42003-021-02700-2

**Published:** 2021-10-13

**Authors:** Chiara Perico, Hongbo Gao, Kate J. Heesom, Stanley W. Botchway, Imogen A. Sparkes

**Affiliations:** 1 School of Biological Sciences, University of Bristol, Life Sciences Building, 24 Tyndall Avenue, Bristol, BS8 1TQ UK; 2 Biosciences, Geoffrey Pope Building, University of Exeter, Exeter, EX4 4QD UK; 3 Bristol Proteomics Facility, University of Bristol, Bristol, BS8 1TD UK; 4 Central Laser Facility, UKRI, Science and Technology Facilities Council, Didcot, OX11 0FA UK; 5 Present Address: Department of Plant Sciences, University of Oxford, South Parks Road, Oxford, OX1 3RB UK; 6 Present Address: Building B-511, School of Agriculture and Biology, Shanghai Jiao Tong University, 800 Dongchuan RD, Minhang District, Shanghai, China

**Keywords:** Cell biology, Myosin, Plant sciences, Plant cell biology, Plant cytoskeleton

## Abstract

Plant cell organelles are highly mobile and their positioning play key roles in plant growth, development and responses to changing environmental conditions. Movement is acto-myosin dependent. Despite controlling the dynamics of several organelles, myosin and myosin receptors identified so far in *Arabidopsis thaliana* generally do not localise to the organelles whose movement they control, raising the issue of how specificity is determined. Here we show that a MyoB myosin receptor, MRF7, specifically localises to the Golgi membrane and affects its movement. Myosin XI-K was identified as a putative MRF7 interactor through mass spectrometry analysis. Co-expression of MRF7 and XI-K tail triggers the relocation of XI-K to the Golgi, linking a MyoB/myosin complex to a specific organelle in *Arabidopsis*. FRET-FLIM confirmed the in vivo interaction between MRF7 and XI-K tail on the Golgi and in the cytosol, suggesting that myosin/myosin receptor complexes perhaps cycle on and off organelle membranes. This work supports a traditional mechanism for organelle movement where myosins bind to receptors and adaptors on the organelle membranes, allowing them to actively move on the actin cytoskeleton, rather than passively in the recently proposed cytoplasmic streaming model.

## Abstract (web summary)

Perico et al. use co-expression analysis and a FRET-FLIM approach to show that the *Arabidopsis* MyoB myosin receptor, MRF7, triggers the relocation of Myosin XI-K to the Golgi. As such, this study provides evidence for plant myosin recruitment and control of organelle movement.

## Introduction

Organelle movement in plant cells relies on the acto-myosin system and plays an important role in plant response to biotic and abiotic stimuli and in directing cell growth and development. In growing root hairs of *Arabidopsis thaliana*, nuclei have been observed to move forward towards the growing end, but maintaining the same distance from the tip^1^. Impairment of nuclear movement by actin depolymerisation or optical trapping suggests that correct positioning of the nucleus is important for coordination of root hair elongation^1^. Chloroplasts and nuclei relocate within the cytoplasm in response to variations in light intensity; chloroplasts reposition from periclinal to anticlinal walls of mesophyll cells (avoidance response) in conditions of high blue light intensities to avoid photodamage^2^. Interestingly, nuclei react similarly to the same light conditions^3^ and one of the proteins involved in the response, phototropin 2 (phot2), also directs the avoidance response for chloroplasts^4,5^, suggesting a movement coordination between the two organelles. More recently, it appears that close association between nuclei and chloroplasts enables ROS mediated retrograde signalling^6^. The speed of the actin-dependent bulk flow movement of cytoplasm, defined as cytoplasmic streaming^7–11^, was observed to correlate with plant and cell size in *A. thaliana*^12^.

Whilst the functional role of movement and repositioning of certain organelles in response to external and internal cues has been partially elucidated, understanding how organelle movement affects plant growth and development still constitutes a challenge. Recent attempts to untangle the functional role of specific organelle movement have focussed on studying and affecting the molecular components driving their movement, particularly class XI myosins and MyoB myosin receptors. Plant myosins from class XI constitute the major contributors to organelle movement^13–18^. *Arabidopsis* encodes 13 class XI myosin members, six of which affect organelle movement. Class XI myosins are structurally and functionally similar to myosins from family V/Myo2p of mammals and yeast:^19,20^ they have a similar length neck region and DIL and PAL domains within the globular tail, suggested to be responsible for cargo binding^21,22^. Functional studies indicate that there is a high degree of complexity and redundancy within the myosin family; one myosin can affect the movement of multiple different types of organelle, and the movement of an organelle can be affected by several myosins^16,17^. Multiple techniques have been employed to reconcile the localisation of myosins XI with their role on organelle movement and plant development, including expression of dominant negative mutants, immunolocalisation, RNAi and knockout of myosin genes. These studies suggested that plant myosins do not tend to localise to specific organelle membranes and that they mostly have overlapping effects on organelle dynamics^16,21–23^ and, in the case of myosin XI-K, XI-1 and XI-2, on cell growth and plant development^13,14^. The one exception is myosin XI-I which is localised to the nuclear envelope and controls movement through interaction with WIT1 and WIT2^24^.

Research has therefore begun to focus on the interface between myosins and organelles, to identify the molecular players involved in myosin recruitment. Recently, two families of *A. thaliana* potential myosin receptors were identified: the MyoB family, constituted of 16 members, and the MadA-B families, four members each^25–27^. Despite differences in their architectures, all MyoB proteins share a predicted myosin-binding Domain of Unknown Function 593 (DUF593) and are present in all land plants^25,28,29^. Interpretation of the role of the MyoB and Mad receptors on specific organelle dynamics has so far proven elusive. With the exception of MadA1, they do not localise to one defined subcellular structure, but rather on unidentified membrane-bounded puncta moving in straight trajectories at an average speed of 3 µm/s^25–27^. The movement and localisation of the MyoB and Mad puncta is myosin-dependent^25,27^. There is evidence that the MyoB2 DUF593 domain is essential for binding to myosin globular tails^25^.

Observations of the effects and localisation of class XI myosins and MyoB receptors in *A. thaliana* resulted in the suggestion of two alternative mechanisms driving organelle movement in *Arabidopsis*, which we will herein be referred to as cytoplasmic streaming-dependent and traditional. In the cytoplasmic streaming-dependent model, MyoB proteins label an entirely new compartment which, due to its high rates of movement, is suggested to be responsible for the generation of cytoplasmic streaming^26^. Organelles such as Golgi, peroxisomes and mitochondria move passively in the wake of the flow generated by the MyoB compartment. Nevertheless, MyoB receptors from other plant systems were observed on specific organelles. Over-expressed *Nt*RISAP localises to the TGN and binds through the DUF593 domain to the pollen tube-specific myosin *Nt*MYOXIpt^29^ and *Zm*Fl1 was immunolocalised on the ER membrane surrounding the protein bodies in maize^28^, indicating that MyoB receptors have the potential to localise to known structures.

In the traditional model, myosin recruitment is driven by a scaffold of receptors and adaptors which assemble hierarchically on the membrane of a specific organelle class. This model is derived in the first instance from observations of myosin V/Myo2p recruitment in mammals and yeast, where the same myosin can be targeted to different organelles via specific protein complexes^30–36^. This model suggests that organelles actively move within the cytoplasm upon myosin recruitment to their membrane.

Here, we provide evidence supporting the traditional model for plant myosin recruitment and control of organelle movement in *A. thaliana*.

## Results

### The MyoB myosin receptor MRF7 localises to moving puncta on the actin cytoskeleton

MyoB proteins are a family of myosin binding proteins present in all land plants^25,28,29^. In *A. thaliana*, the MyoB family is composed of 16 members divided into six subgroups on the basis of their architectures^25^. Despite a variable domain composition between subgroups, all MyoB proteins share a predicted myosin-binding Domain of Unknown Function 593 (DUF593). Initial characterisation identified a MyoB receptor which appeared to contain a truncated DUF593 region^25^; more recent annotations established a variant with the complete DUF593 region. We focussed our attention on the full-length variant which we will herein refer to as Myosin Recruitment Factor 7 (MRF7). MRF7 is a 324 amino acids long putative myosin receptor belonging to subgroup IIA of the *At*MyoB family^25^. It does not appear to have any predicted trans-membrane domains or motifs for lipid anchoring and can therefore be considered a soluble protein. The potential myosin-binding DUF593 domain spans the 112 N-terminal amino acids.

**Fig. 1:**
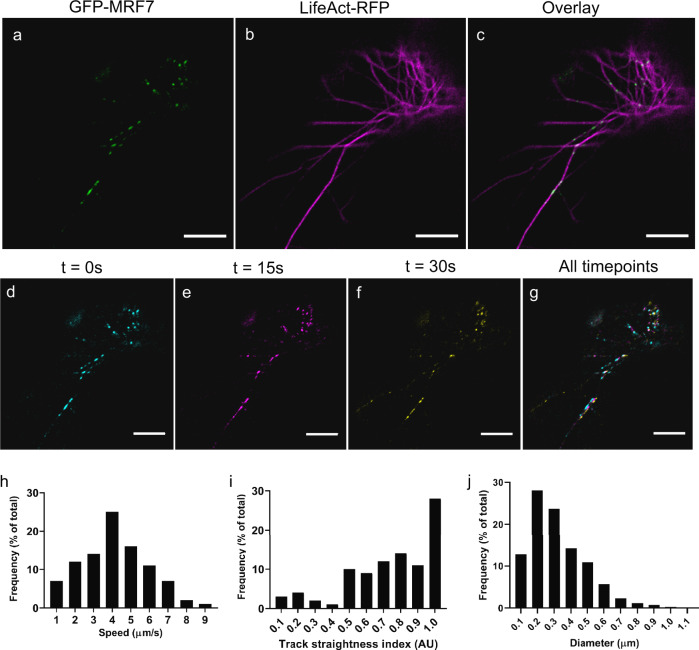
GFP-MRF7 moves on discrete puncta along the actin cytoskeleton. GFP-MRF7 localises to discrete puncta in a beads-on-a-string configuration (**a**). Co-expression with the actin marker LifeAct-RFP (**b**, **c**) shows that GFP-MRF7 moves along actin tracks. The false colour timelapse images in panels **d**–**g** show the movement of GFP-MRF7 puncta: cyan (**d**), magenta (**e**) and yellow (**f**) panels represent successive still images taken at 15 s intervals. Panel **g** is the overlay of all timepoints: white pixels depict static regions. Distributions in panels **h** and **i** describe the movement characteristics of the GFP-MRF7 puncta, specifically the frequency distributions of speed (**h**) and track straightness index (**i**) where a track straightness index of 1 indicates a straight trajectory. All images collected 2 days post-infiltration from tobacco leaf epidermal cells (*n* = 95, 13 cells, 2 independent experiments). **j** Frequency distribution of the GFP-MRF7 puncta diameters measured with the Analyze Particle tool in ImageJ (*n* = 689, 30 cells, 3 independent experiments). Scale bar = 10 µm.

Initially, we sought to determine whether MRF7 displayed similar characteristics to other members of the *At*MyoB family^25,27^. Transient over-expression of GFP-MRF7 in tobacco leaf epidermal cells revealed a typical MyoB localisation to numerous discrete puncta^25,27^ (Fig. 1). The straightness of the trajectories followed by the puncta suggested they could be moving on cytoskeletal tracks. Co-expression of GFP-MRF7 with the actin marker LifeAct-RFP in tobacco leaf epidermal cells, indicated that the puncta collocate to (Fig. 1a–c) and move along (Fig. 1d–g), the actin. This result is indicative of MRF7 puncta association with actin filaments (Fig. 1a–c, Supplementary Fig. 1a–c). Furthermore, GFP-MRF7 puncta movement ranged from 1–9 μm/s with an average speed of 4 μm/s (Fig. 1h, Supplementary Fig. 2, Supplementary Data 1), are similar to those presented for other MyoB receptors^26^, with the highest speeds corresponding to the maximum reported speeds of cytoplasmic streaming in *A. thaliana*^37^.Track straightness index, which provides a quantification on the directionality of the tracks (Fig. 1i, Supplementary Fig. 2, Supplementary Data 1) supports the initial observation of puncta following straight trajectories. GFP-MRF7 puncta diameter ranged from 0.1 µm to 1 µm with the majority being less than 0.5 µm (Fig. 1j, Supplementary Data 1), is compatible with the average diameters of other MyoB proteins^25,27^.

MRF7 localisation to motile puncta therefore displays similar characteristics to other *At*MyoB family members.

### MRF7 localises to Golgi and affects Golgi speed

**Fig. 2:**
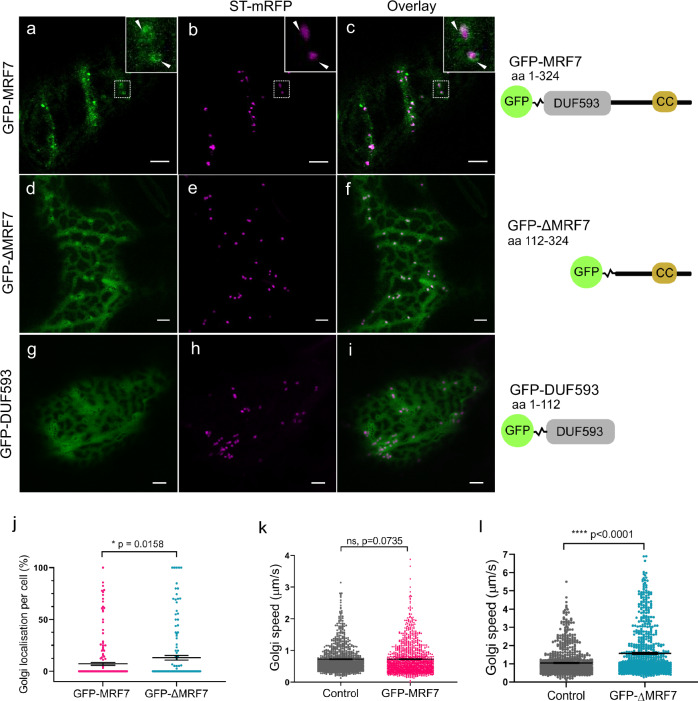
DUF593-independent Golgi localisation of MRF7 and effects on Golgi movement. Transient expression of full-length and truncated MRF7 (green) with the Golgi marker ST-mRFP (magenta) in tobacco leaf epidermal cells. Schematics on the right hand-side of each panel show the architecture of the truncations used in this study; DUF593 Domain of Unknown Function 593, CC coiled-coil. **a**–**c** In addition to the punctate localisation shown in Fig. 1, GFP-MRF7 exhibits Golgi surface localisation. Smaller panels in the right hand-side provide a magnification of the region indicated by the dotted square. Arrowheads indicate the Golgi surface localisation of GFP-MRF7. **d**–**f** Absence of the DUF593 domain does not impair the Golgi localisation of MRF7. **g**–**i** The DUF593 domain itself is cytosolic and does not colocate to Golgi. **j** Significant increase in Golgi localisation in cells expressing GFP-ΔMRF7 compared with GFP-MRF7. Each datapoint represents the percentage of Golgi in a particular cell. Mann–Whitney test, data collected from 207 (GFP-MRF7) and 143 (GFP-ΔMRF7) cells across at least 16 independent infiltrations. Bars represent mean ± SEM. Golgi speed was analysed in cells expressing Golgi marker and either GFP-MRF7 (**k**) or GFP-ΔMRF7 (**l**). Bars represent mean ± SEM. GFP-MRF7 does not significantly influence Golgi movement (*n* = 1185, 40 cells, 4 independent experiments) when compared to control cells expressing Golgi marker alone (*n* = 1000, 40 cells, 4 independent experiments). GFP- ΔMRF7 significantly increases Golgi speed (*n* = 717, 36 cells, 5 independent experiments) when compared to control cells expressing Golgi marker alone (*n* = 855, 42 cells, 5 independent experiments). Mann–Whitney test. All imaging carried out in tobacco leaf epidermal cells two days post-infiltration. Scale bar = 5 μm.

In addition to its more traditional MyoB punctate localisation, further observations in tobacco revealed that GFP-MRF7 localised to the Golgi surface (white arrowheads, Fig. 2a–c, Supplementary Fig. 1a–c). The current literature on *A. thaliana* MyoB receptors lacks evidence for MyoBs collocating to a known organelle whose movement they affect^25–27^. Therefore, the MRF7 Golgi localisation represents, to the best of our knowledge, the first example of specificity of MyoBs in *A. thaliana*.

The DUF593 domain potential role in MRF7 localisation to Golgi was investigated through over-expressing the domain itself (GFP-DUF593) or MRF7 lacking the domain (GFP-ΔMRF7). GFP-ΔMRF7, localises to the Golgi (Fig. 2d–f), whereas GFP-DUF593 is cytosolic (Fig. 2g–i). indicating that the Golgi targeting information is not contained within the DUF593 region. The most striking effect of the lack of the DUF593 domain is linked to the disappearance of the MyoB puncta; GFP-MRF7 locates to Golgi/puncta/cytosolic (Fig. 2a–c), GFP-ΔMRF7 only displays a Golgi/cytosolic pattern (Fig. 2d–f). This could indicate that DUF593 is linked to the formation or perhaps the correct functioning of the puncta, despite GFP-DUF593 not directly localising to punctate structures by itself. Quantification of the percentage of GFP-MRF7 and GFP-ΔMRF7 per cell localising on Golgi revealed that not all the Golgi in the same cell display the proteins on their surface (Fig. 2j, Supplementary Data 2). Occasionally all the Golgi will display MRF7, but more frequently only some of them will have MRF7 on the surface, indicative of a heterogeneous Golgi-MRF7 population within the cell. The quantification also highlighted that a significantly higher percentage of GFP-ΔMRF7 per cell localise to the Golgi compared to GFP-MRF7 (7.2% ± 1.3%, and 13.3% ± 2.2%, respectively). This could indicate that lack of the DUF593 domain triggers a more stable Golgi localisation. GFP-MRF7 expression does not influence Golgi movement compared to the control (both 0.72 ± 0.01 µm/s, Fig. 2k, Supplementary Fig. 3a, Supplementary Data 2). GFP-ΔMRF7 however significantly increases Golgi speed when compared to the control (Figs. 2l, 1 ± 0.02 µm/s versus 1.5 ± 0.05 µm/s, Supplementary Fig. 3b, Supplementary Data 2).

**Fig. 3:**
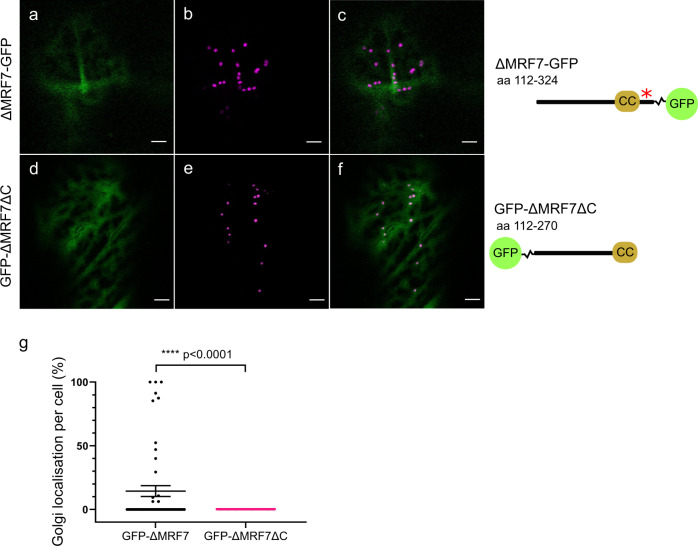
MRF7 C-terminal region affects Golgi localisation. **a**–**c** C-terminal fusion of GFP to ΔMRF7 localises to the cytosol, suggesting that the Golgi-targeting information could reside at the C-terminus of MRF7 (red asterisk). The C-terminal GFP fusion could be responsible for masking such signal, preventing a Golgi localisation. **d**–**f** Localisation of GFP-ΔMRF7ΔC reflects that of ΔMRF7-GFP, indicating that the last 54 amino acids are fundamental to target MRF7 to the Golgi. **g** Quantification of GFP-ΔMRF7ΔC Golgi localisation compared to that of GFP-ΔMRF7. Each datapoint represents one cell; values on the left-hand side indicate the percentage of Golgi in a particular cell with GFP-ΔMRF7 or GFP-ΔMRF7ΔC surface localisation. Data from 53 cells (GFP-ΔMRF7) and 51 cells (GFP-ΔMRF7ΔC) from 5 independent experiments, Mann–Whitney test, ****P < 0.0001. All imaging carried out two days post-infiltration. Scale bar = 5 μm.

Unlike GFP-ΔMRF7, C-terminally tagged ΔMRF7, ΔMRF7-GFP (Fig. 3a–c) does not locate to the Golgi indicative of a potentially masked targeting domain within the C terminus by the GFP tag (red asterisk, Fig. 3a–c). Comparison between GFP-ΔMRF7 and GFP-ΔMRF7ΔC, construct lacking the last 54 C-terminal amino acids downstream of the coiled-coil domain (Fig. 3d–f), indicates that the C-terminal region is essential for Golgi targeting; GFP-ΔMRF7 locates to Golgi unlike GFP-ΔMRF7ΔC which is cytosolic (Fig. 3g, Supplementary Data 2).

**Fig. 4:**
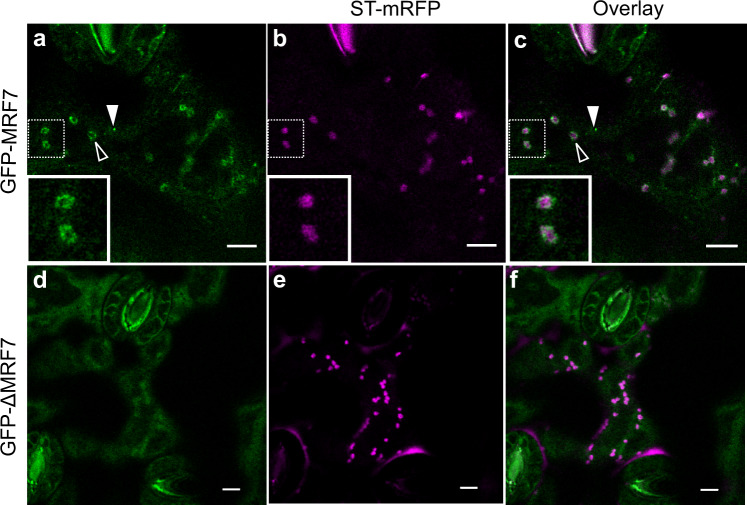
Localisation of GFP-MRF7 and GFP-ΔMRF7 in *A. thaliana* is consistent with that observed in tobacco. *A. thaliana* lines stably over-expressing GFP-MRF7 (green) or GFP-ΔMRF7 (green) were generated by floral dipping of wild-type Col-0 plants. Stable lines from T4 generation were then crossed with a ST-mRFP (magenta) marker line. Progeny were imaged 14 days post-germination. **a**–**c** As in tobacco leaf epidermal cells (Fig. 2a–c), GFP-MRF7 localises both to puncta (filled arrowheads) and to the Golgi surface (empty arrowheads) in Arabidopsis leaf epidermal cells. Magnification in the top panel shows a detail of the Golgi localisation: GFP-MRF7 fluorescence is brighter on subdomains of the Golgi. **d**–**f** As in tobacco leaf epidermal cell (Fig. 2d–f) GFP-ΔMRF7 localises to the Golgi and in the cytosol in Arabidopsis leaf epidermal cells. Scale bar = 5 µm.

Comparison of localisation patterns of GFP-MRF7 and GFP-ΔMRF7 in tobacco and *Arabidopsis* leaf epidermal cells indicate that the heterologous system is consistent with the native plant species: GFP-MRF7 localises to puncta (Fig. 4a, white arrowheads), cytosol and is present on a subpopulation of Golgi (Fig. 4a, empty arrowhead) and concentrated on certain regions of the Golgi in stable *Arabidopsis* lines; GFP-ΔMRF7 localised to Golgi and cytosol, there are no puncta in stable *Arabidopsis* lines (Fig. 4b).

### Identification of myosin XI-K as MRF7 binding partner

The presence of MRF7 on the Golgi represents, to the best of our knowledge, the first example of an *A. thaliana* MyoB receptor on one specific organelle. Pull downs from stably expressing *A. thaliana* lines were carried out in an attempt to isolate a potential myosin interactor. Given that the DUF593 domain is responsible for myosin binding^25,27,29^, comparisons between pull downs from both full length (GFP-MRF7) and DUF593 truncated lines (GFP-ΔMRF7) were performed.

Supplementary Fig. 4 indicates chimeric protein expression in the transgenic lines, and presence of the fusions in the pull-down assay prior to liquid chromatography–mass spectrometry (LC–MS) analysis. Myosin XIK was isolated in pull downs from the GFP-MRF7 samples but was absent from the GFP-ΔMRF7 samples (Supplementary Data 3). These results are indicative of the DUF593 domain within MRF7 being essential for the interaction with myosin XIK.

### MRF7 triggers the recruitment of myosin XI-K tail to the Golgi surface

XI-K tail was previously shown to be present in the cytosol and on motile puncta, and to affect the movement of multiple organelles including Golgi, peroxisomes, mitochondria and the ER^15,16,38^. It was not observed to localise to the Golgi in a similar manner to MRF7. We tested whether expression of GFP-MRF7 could influence myosin XI-K subcellular localisation, and whether the effect might be DUF593-dependent. To this end, myosin XI-K localisation pattern was monitored in the presence and absence of GFP-MRF7 and GFP-ΔMRF7 in tobacco leaf epidermal cells. We chose to over-express a motor less XI-K construct, myosin XI-K tail^16^, rather than the full length myosin, as the localisation and effects of XI-K tail on organelle dynamics, including Golgi, has already been documented^15,16^.

**Fig. 5:**
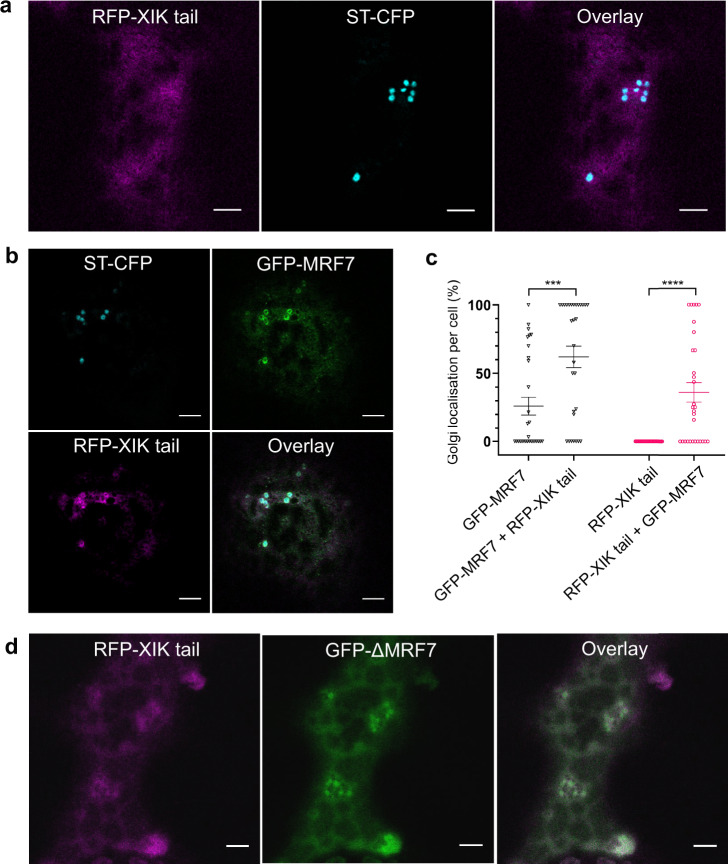
Myosin XI-K tail relocates from the cytosol to the Golgi surface in the presence of GFP-MRF7. **a** Myosin RFP-XIK tail is present in the cytosol when coexpressed with the Golgi marker (StCFP, cyan). **b** Myosin RFP-XIK tail (magenta) is present on the Golgi surface and in the cytosol when coexpressed with GFP-MRF7 (green) and the Golgi marker (StCFP cyan), see overlay image. **c** Quantification of the GFP-MRF7 (black datapoints) and RFP-XIK (magenta datapoints) tail Golgi localisation per cell, when expressed with Golgi marker only, or when expressed with each other and the Golgi marker. RFP-XIK tail significantly increases the Golgi localisation of GFP-MRF7 (black datapoints), similarly, GFP-MRF7 significantly increases Golgi localisation of RFP-XIK tail (magenta datapoints). The analysis was performed on 30 cells across three independent experiments; each datapoint represents one cell (mean ± SEM), ***p < 0.001, ****p < 0.0001, Mann–Whitney U test. **d** Myosin RFP-XIK tail (magenta) remains in the cytosol when coexpressed with GFP-ΔMRF7 (green), see overlay image. All constructs were transiently expressed in tobacco leaf epidermal cells and imaging was performed two days post-infiltration. Scale bar = 5 µm.

Consistent with previous results^16^, RFP-XIK tail was observed to be cytosolic or occasionally localised to unidentified puncta in the absence of either GFP-MRF7 or GFP-ΔMRF7 (Fig. 5a). In the absence of RFP-XIK tail, GFP-MRF7 and GFP-ΔMRF7 maintained the localisation pattern shown in Fig. 2a–c and Fig. 2d–f, respectively. Upon co-expression with GFP-MRF7, myosin XI-K tail collocates with the receptor and labels circular Golgi-like structures; this effect was not observed upon co-expression of RFP-XIK tail with GFP-ΔMRF7 (Fig. 5d). Therefore, XIK localisation is affected through expression of the full length MRF7 which is dependent on the DUF593 region within MRF7.

Through triple co-expression of GFP-MRF7, RFP-XIK tail and the Golgi marker ST-CFP, we were able to determine that GFP-MRF7 and RFP-XIK tail indeed co-locate to the surface of Golgi bodies (Fig. 5b). MRF7 therefore localises to the Golgi and in turn could ‘trigger’ the recruitment of XIK to the Golgi. To quantify the recruitment process, we determined the percentage of Golgi bodies displaying RFP-XIK tail per cell area in cells expressing RFP-XIK tail alone, and in cells co-expressing both RFP-XIK tail and GFP-MRF7 (Fig. 5c, magenta). While initially RFP-XIK tail is solely cytosolic (Fig. 5a), in the presence of GFP-MRF7 an average of 36% of Golgi bodies per cell area display XI-K tail (Fig. 5c, *p* < 0.001). MRF7 therefore has a significant effect on XIK tail localisation and triggers a change in localisation from the cytosol to the Golgi, indicative of MRF7 acting as a recruitment factor for XIK to Golgi. We carried out the reciprocal quantification to determine whether XIK tail affected MRF7 Golgi localisation. RFP-XIK tail expression significantly increases Golgi localised GFP-MRF7; increase from 26 to 62% upon RFP-XIK tail expression, Fig. 5c, black, *p* = 0.0005). In addition, we noticed that co-expression of GFP-MRF7 and RFP-XIK tail also appeared to cause the disappearance of the MRF7 puncta (Fig. 5b). Percentages of Golgi bodies displaying GFP-MRF7 and RFP-XIK tail are reported in Supplementary Tables 2 and 3 and Supplementary Data 4.

### In vivo interaction of MRF7 and myosin XI-K tail

Mass spectrometry and co-expression studies suggest that MRF7 interacts with myosin XI-K and that the interaction is DUF593-dependent. FRET-FLIM (Fӧrster Resonance Energy Transfer - Fluorescence Lifetime Imaging Microscopy) analyses were carried out to test whether the co-localisation of MRF7 and XI-K tail is the result of a direct in vivo interaction^39^. FRET is a photophysical phenomenon in which energy is non-radiatively transferred from an excited donor to a non-excited quencher (acceptor) within a distance of approximately 10 nm^40^. The FRET determined by excited state lifetime measurements can be affected by several factors including the close proximity of other molecules due to dipole-dipole interactions. One method employed to quantify FRET is FLIM: upon occurrence of FRET, a reduction of the excited state lifetime (τ) of the donor molecule is observed. Measurement of the donor’s lifetime in the presence and absence of an acceptor can give an indication of whether the two molecules are interacting. FRET-FLIM is now widely applied as a method of choice for live cell quantification of molecular interactions and protein-protein interactions across viruses, plant and mammalian cells^41,42^.

**Fig. 6:**
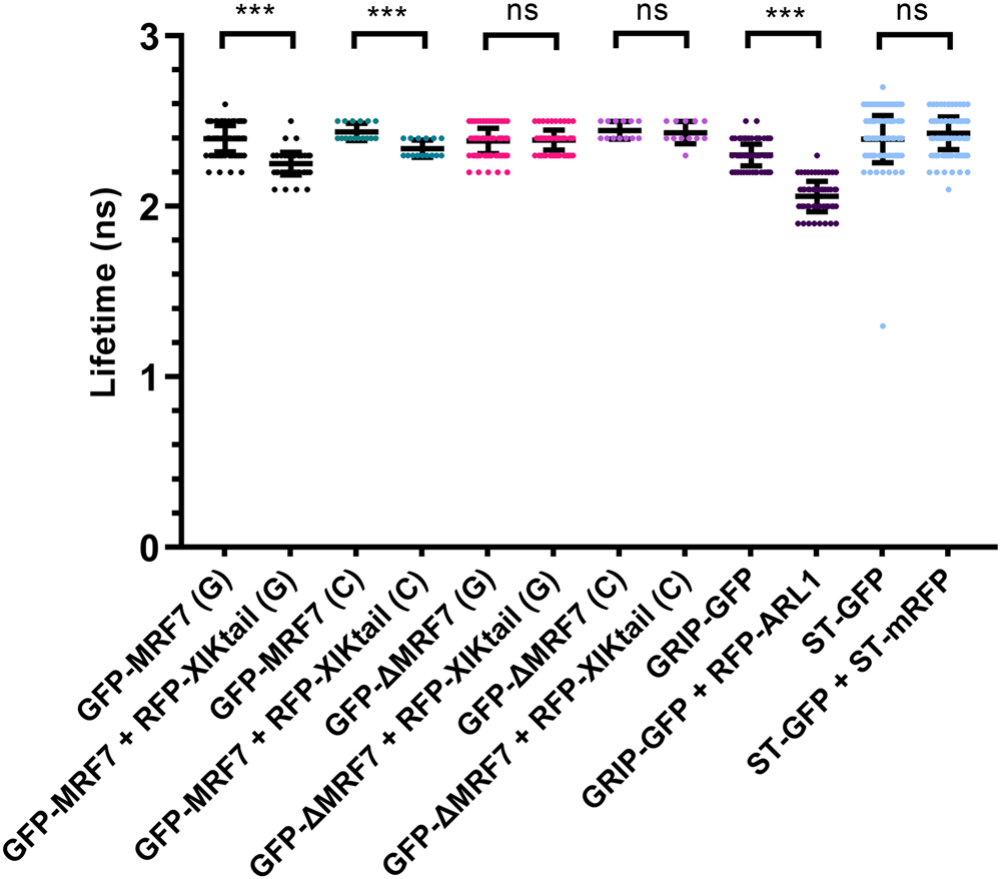
FRET-FLIM indicates that GFP-MRF7 interacts in vivo with myosin XIK tail via its DUF593 domain. GFP lifetime values (mean ± SD) in nanoseconds (ns) for denoted constructs transiently expressed in tobacco leaf epidermal cells are shown. GFP fusions were expressed by themselves and their lifetimes compared when coexpressed with a potential interactor. A significant drop in lifetime upon coexpression is indicative of interaction. The labels G and C indicate whether the lifetime was measured on Golgi or in the cytosol, respectively. ST-GFP and ST-RFP constitute the negative control (i.e., no significant drop in GFP lifetime indicating no interaction), GRIP-GFP and RFP-ARL1 are the positive control (i.e. significant drop in lifetime indicating an interaction). The lifetime for the positive and negative control FRET pairs were measured on Golgi. There is a significant drop in lifetime in both the cytosol and Golgi when GFP-MRF7 is coexpressed with RFP-XIK tail indicating that both fusions interact in the cytosol and on the Golgi surface. There is no significant drop in lifetime when GFP-ΔMRF7 is coexpressed with RFP-XIK tail indicating that the DUF583 region is required for the interaction between MRF7 and XIK-tail. ***p < 0.001, ns non-significant, Student’s t-test. Sample size and number of independent experiments are indicated in Supplementary Tables 1 and 2.

Here, RFP-XIK tail (quencher) was transiently coexpressed with either GFP-MRF7 or GFP-ΔMRF7 (donor molecules) in tobacco leaf epidermal cells and interaction assessed for the cytosol and Golgi localised populations. Due to the high rates of movement of Golgi bodies in plant cells, Latrunculin B treatment was necessary for appropriate data collection from the Golgi surface^43,44^. GFP-MRF7 interacts with mRFP-XIK tail in the cytosol and on the Golgi; the donor lifetime, GFP-MRF7, significantly reduces upon coexpression with acceptor, RFP-XIK tail (2.4 ± 0.1 ns versus 2.3 ± 0.1 ns respectively, Fig. 6, Table 1, the 0.1 ns reduction in lifetime was determined to be statistically significant, *p* < 0.001). A Δτ = 0.1 ns for the GFP-MRF7 + RFP-XIK tail pair was determined to correspond to a 4.2% FRET efficiency and to an estimated intermolecular distance of 9.1 nm (Table 1). The relatively low FRET efficiency and the estimated distance between GFP-MRF7 and RFP-XIK tail indicates that the interaction probably takes place over a relatively large spatial separation, although within the ~ 10 nm range for FRET to happen. The distance could be indicative either of an additional protein within the complex or reflect experimental spatial differences from the position of the fluorophore relative to the regions within the proteins where interaction occurs. No interaction was detected between GFP-ΔMRF7 and RFP-XIK tail on the Golgi or in the cytosol; GFP lifetime remained at 2.4 ± 0.1 ns (Fig. 6, *p* > 0.999). Note, as previously described, the Golgi localised proteins GRIP-GFP and RFP-ARL1 were used as the positive control and the Golgi marker ST-GFP and ST-mRFP co-expression acted as negative control^43^. A summary of FRET pairs, lifetime values and sample sizes used in this study is provided in Table 1, Supplementary Data 5 and Supplementary Table 1.

**Table 1:** Average lifetime values, FRET efficiencies and estimated intermolecular distance for the FRET pairs tested.

FRET pair	*τ*_D_ (ns)	*τ*_DA_ (ns)	*E*_FRET_ (%)	*r* (nm)
GFP-MRF7 + RFP-XIK tail ^(*)^	2.4 ± 0.1	2.3 ± 0.1	4.2	9.1
GFP-ΔMRF7 + RFP-XIK tail ^(*)^	2.4 ± 0.1	2.4 ± 0.1	—	» 10
GRIP-GFP + RFP-ARL1	2.3 ± 0.1	2.1 ± 0.1	8.7	7.9
ST-GFP + ST-mRFP	2.4 ± 0.1	2.4 ± 0.1	—	» 10

τ_D_ indicates the donor’s lifetime in the absence of the quencher ± SD, τ_DA_ indicates the donor’s lifetime in the presence of the quencher ± SD. Asterisks indicate combinations whose lifetimes were measured both on Golgi and in the cytosol. FRET efficiencies (*E*_FRET_) and estimated intermolecular distances (r) were calculated according to Eqs. (1) and (2) described in the Methods section.

Altogether, these results indicate that MRF7-XIK tail interaction is direct (or within the 10 nm range), and that the predicted myosin binding domain DUF593 mediates the in vivo interaction between MRF7 and XI-K tail on the Golgi surface. Moreover, the interaction between MRF7 and XI-K can also occur in the cytosol, potentially driving XI-K recruitment to the Golgi. Results do not exclude the possibility that the complex may shuttle on and off the Golgi.

### Effect of MRF7 on other myosin tails localisation

FRET-FLIM confirmed the DUF593-dependent interaction between MRF7 and XI-K tail. Although other myosins were not detected in mass spectrometry analysis as potential MRF7 interactors, it is known that some class XI myosins other than XI-K can influence Golgi movement^13–16^ and that the myosin XI and MyoB networks are highly redundant^27^. Pairwise yeast-2-hybrid screens (Y2H) have shown that MyoB receptors interact with multiple myosins from class XI and myosins from class XI interact with multiple MyoB receptors^27^. To test whether the effects of MRF7 expression on myosin localisation are specific for myosin XI-K, GFP-MRF7 was co-expressed with myosin XI-1 and XI-A tails^16^ and their Golgi localisation was quantified. The choice of XI-1 and XI-A tails was based on the fact that the former was implicated in Golgi movement and that the GT2 domain within its tail was shown to localise to Golgi^16,23^. The latter instead does not appear to exert any effect on Golgi dynamics^16^.

**Fig. 7:**
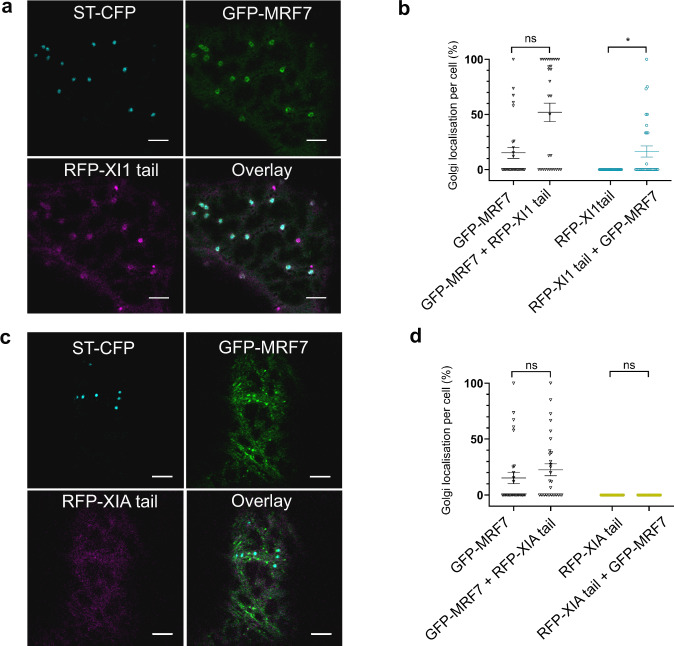
Effects of GFP-MRF7 expression on myosin XI-1 and XI-A tails localisation. **a** RFP-XI1 tail (magenta) localises to the Golgi in the presence of GFP-MRF7 (green). ST-CFP (cyan) labels the Golgi stacks. **b** Quantification of the Golgi localisation of GFP-MRF7 (black datapoints) and RFP-XI1 tail (cyan datapoints) when expressed with Golgi marker only, or following co-expression with each other and the Golgi marker. RFP-XI-1 tail significantly increases the Golgi localisation of GFP-MRF7 (black datapoints), similarly, GFP-MRF7 significantly increases Golgi localisation of RFP-XI-1 tail (cyan datapoints). **c** RFP-XIA tail (magenta) does not re-localise to the Golgi in the presence of GFP-MRF7 (green). ST-CFP (cyan) labels the Golgi stacks. **d** Quantification of the Golgi localisation of GFP-MRF7 (black datapoints) and RFP-XIA tail (yellow datapoints) when expressed with Golgi marker only, or following co-expression with each other and the Golgi marker. RFP-XIA tail does not significantly increase the Golgi localisation of GFP-MRF7 (black datapoints). GFP-MRF7 does result in RFP-XIA tail (magenta datapoints) localisation to the Golgi. **b**, **d** Each datapoint representing one cell (mean ± SEM), *n* = 30, three independent infiltrations. Mann–Whitney U test, **p = 0.002, ***p = 0.0008, ns non-significant. The constructs were transiently expressed in tobacco leaf epidermal cells and imaging was performed two days post-infiltration. All scale bars = 5 µm.

In the absence of MRF7, XI-1 (Supplementary Fig. 5a) and XI-A (Supplementary Fig. 5b) tails are distributed in the cytosol and occasionally on puncta^16^. Co-expression of GFP-MRF7 with RFP-XI1 tail leads to a significant redistribution of RFP-XI1 tail from the cytosol to the Golgi (Fig. 7a, b cyan datapoints, *p* = 0.0008). RFP-XI1 tail can also significantly increase GFP-MRF7 on the Golgi (Fig. 7b, black datapoints *p* = 0.002), similarly to the effect observed for XI-K tail (Fig. 5c).

No significant differences in GFP-MRF7 or myosin XI-A tail localisation were detected upon co-expression (Fig. 7c and Supplementary Fig. 5b). The average percentage of Golgi displaying GFP-MRF7 does not change before or after co-expression with XI-A tail (Fig. 7c, *P* = 0.1645) and myosin XI-A tail remains cytosolic despite co-expression with GFP-MRF7 (Fig. 7c, *P* > 0.999). Percentages of Golgi bodies displaying GFP-MRF7, RFP-XI1 and RFP-XIA tails are reported in Supplementary Tables 2 and 3 and Supplementary Data 4.

Altogether, these results suggest that myosin tails and receptors can exert a mutual effect on each other’s localisation. In particular, GFP-MRF7 causes a relocation to the Golgi of the myosin tails involved in Golgi movement, XI-K and XI-1 in this instance. Moreover, the presence of myosin tails involved in the regulation of Golgi movement increases GFP-MRF7 on the Golgi. No such effect was observed upon co-expression of GFP-MRF7 with RFP-XIA tail, a myosin tail not involved in regulating Golgi movement^16^. We propose that MRF7 may work as a universal adaptor for myosin recruitment specifically to the Golgi.

## Discussion

**Fig. 8:**
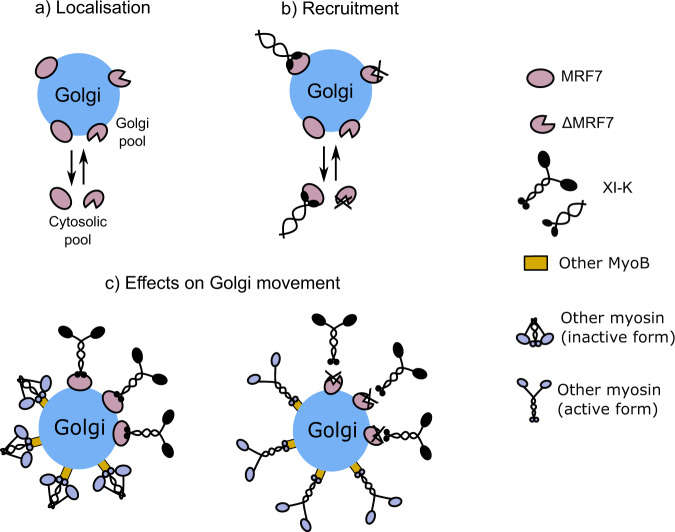
Suggested model for MRF7 Golgi localisation, myosin recruitment and effects on Golgi movement. **a** MRF7 and its N-terminally truncated version, ΔMRF7, localise to the Golgi surface and in the cytoplasm. As a soluble protein, this suggest that MRF7 could have the ability to cycle on and off the Golgi surface. The MRF7 Golgi localisation was determined to be driven by its C-terminal domain. **b** The DUF593 domain is essential for myosin recruitment and binding. As the interaction between XI-K tail and MRF7 takes place both on the Golgi membrane and in the cytosol, we suggest that the interaction could initially take place in the cytosol and trigger XI-K recruitment to the Golgi. **c** Effects of GFP-MRF7 and GFP-ΔMRF7 over-expression on Golgi movement. Golgi movement is preferentially driven by myosin XI-K (black). Other MyoB receptors could be associated to the Golgi and bind myosins kept in the inactive form (blue). Deletion of the DUF593 domain impairs the binding of MRF7 to XI-K; in this scenario the other MyoB receptor would be responsible for driving Golgi movement.

Here we provide evidence that *A. thaliana* class XI myosins can be targeted to a specific organelle upon co-expression with their receptor/adaptor protein. We identified an *At*MyoB receptor, MRF7, which collocates to the Golgi surface alongside the more typical MyoB punctate localisation. Expression of N and C terminal GFP tagged full length and truncated variants of MRF7 indicate that the targeting region is present at the C terminus. Interaction between MRF7 and XI-K is dependent on the N terminal DUF593 region which is not masked in the GFP-MRF7 fusion. Pull down experiments indicated myosin XI-K as a MRF7 binding partner, and co-expression of MRF7 and XI-K tail in tobacco leaf epidermal cells triggered the Golgi surface localisation of myosin XI-K tail. FRET-FLIM analysis revealed that the re-localisation of myosin XI-K tail from the cytosol to the Golgi surface is likely a consequence of the direct in vivo interaction between MRF7 and XI-K tail via the MRF7 DUF593 domain. Furthermore, we suggest that MRF7 could act as a universal myosin adaptor for the Golgi apparatus, due to its ability to induce Golgi re-localisation of myosins involved in the control of Golgi dynamics. Our results link a MyoB:myosin XI complex to one specific organelle class in *A. thaliana* and shed light on the mechanisms driving organelle movement in plant cells. Schematic model depicting MRF7 localisation, myosin recruitment and effects on Golgi movement is provided (Fig. 8).

Similar to other reported MyoB receptors, GFP-MRF7 localises to unknown motile puncta which display similar characteristics. The lowest registered speeds are consistent with the velocity of organelles such as Golgi and peroxisomes, while the average speed reflects that of other MyoB receptors^26^. Notably, the highest speeds registered for the GFP-MRF7 puncta are slightly higher or comparable to the speed of cytoplasmic streaming in *A. thaliana*^12^. Due to their association to membranous structures^25^, *A. thaliana* MyoBs were suggested to constitute a completely new compartment that is the main driving force of cytoplasmic streaming, with organelles moving passively in the wake^26^. However, mathematical models in *Chara corallina* suggest that structures with the diameter of MyoB particles would have to move at twice the speed of cytoplasmic streaming to sustain the passive movement of organelles within the cytoplasm^25–27,45^.

Because of their membranous nature, it was speculated that the MyoB puncta could also belong to sub-domains of organelle membranes. Here we show that MRF7 can localise both to unknown punctate structures and to the Golgi, linking an *Arabidopsis* MyoB receptor to a specific organelle class. This localisation pattern differs from that of other MyoB receptors described so far in *A. thaliana*, but organelle-specific localisation was previously observed for the MyoB proteins *Nt*RISAP on tobacco TGN^29^ and *Zm*Fl1 on the endoplasmic reticulum surrounding protein bodies in maize^28^. Notably, the other reported *At*MyoBs were expressed in *A. thaliana* cells under the control of their native promoters^25,27^, whereas GFP-MRF7 and *Nt*RISAP were over-expressed. At present, the stoichiometry of a functional myosin:myosin receptor complex on organelle membranes is unknown. It is tempting to speculate that myosins and MyoB copy number on organelle membranes could be tightly regulated to promote the fine tuning of organelle positioning. Here, low numbers of the complex required to generate movement could be below the detection limits of conventional confocal microscopy, resulting in difficulty in deciphering the mechanism behind myosin recruitment. Therefore, using an over-expression system could help overcome the difficulties of imaging a fluorescently tagged *At*MyoB on an organelle membrane under native conditions, and could be used to identify the components of the myosin-receptor complexes.

To verify that the Golgi localisation was not the result of heterologous expression, we generated stable *A. thaliana* lines over-expressing GFP-MRF7 and GFP-ΔMRF7. The localisation pattern of both chimeras reflect that observed in tobacco, thus ruling out a species-specific effect on localisation. We also observed that, in both plant species, GFP-MRF7 is occasionally more concentrated on specific Golgi domains, both in tobacco and in *A. thaliana* (magnifications in Fig. 2a–c and Fig. 4a–c). This characteristic has been observed previously for GNOM and GNL1^46^.

Furthermore, we investigated the contribution of various domains of MRF7 on Golgi targeting. Expression of GFP-DUF593 indicates that the myosin binding domain is not essential for targeting to the Golgi (Fig. 2d–i). C-terminal GFP tagging of ΔMRF7 results in a cytosolic localisation (Figs. 3a–c, g) whereas GFP-ΔMRF7 localises to the Golgi (Fig. 2d–f). Altered subcellular localisation following GFP tagging of proteins at either the N- or C-terminus is not uncommon: *At*DHAR3, a chloroplastic dehydroascorbate reductase, localises to *Arabidopsis* chloroplasts when expressed as GFP-DHAR3, but to the cytosol when expressed as DHAR3-GFP^47^. The observations were consistent with DHAR3 having a predicted N-terminal plastid signal peptide, which the GFP tag was most likely masking in the fully folded GFP-DHAR3 chimera^47^. Confirmation of the MRF7 C terminal regions role in Golgi targeting was indicated through the cytosolic localisation of the GFP-ΔMRF7ΔC, which lacks both the DUF593 domain and the C terminal 54 amino acids. The shift in the subcellular localisation pattern (Fig. 3g) is indicative of a targeting signal or domain within the 54 C-terminal amino acids being essential for Golgi localisation, however it does not preclude that regions, such as a coiled coil domain, may facilitate in this process.

Mass spectrometry data indicated that MRF7 can interact with myosin XI-K via its DUF593 domain. To determine whether the simultaneous expression of MRF7 and myosin XI-K could trigger an effect on the two proteins localisation, we co-expressed GFP-MRF7 and the globular tail domain of the myosin, RFP-XIK tail^16^, in tobacco leaf epidermal cells. At physiological calcium concentrations (Ca^2+^), myosin V/Myo2p adopts two different conformations, folded and extended. In the absence of a bound cargo, an intramolecular interaction between the globular tail and the motor domain takes place; as a result, the tail domain folds onto the motor domain inhibiting its ATPase activity, and the myosin is considered as mechanically and enzymatically “switched off”^48–50^. In the presence of a bound cargo, the head-to-tail interaction is disrupted, and the myosin molecule adopts the more familiar “extended” conformation to drive the movement of the cargo to which it is bound^48^. It is tempting to speculate that a full-length *Arabidopsis* myosin could cycle on and off its target organelle by switching from a folded to an extended conformation and vice versa. Given the considerable similarities with myosin V^19,20^, it is quite likely that a similar regulation could also take place for myosins from class XI, and has been proposed for XI-1 and XI-K^23,51^. Taking into account the considerations regarding myosin copy number and ATPase activity regulation, the expression of the full-length myosin XI-K, even under over-expression conditions, may not be sufficient to allow a significant population to accumulate on the organelle to enable detection using conventional confocal microscopy. The motor-less myosin XI-K^16^ is most-likely unable to adopt a folded conformation (as it lacks the motor domain) and we speculate that this could lead to the truncated protein to be permanently “attached” to its receptor on the organelle. Moreover, over-expression of XI-K tail alone could lead to the saturation of the endogenous MRF7, complicating the XI-K tail detection on a target organelle (Fig. 5a). By artificially increasing the GFP-MRF7 pool through over-expression, its levels could match those of XI-K tail, effectively targeting all or most of the available XI-K tail to the Golgi, enhancing the population to detectable levels (Fig. 5b). Triple expression with the Golgi marker ST-CFP confirmed that the full-length GFP-MRF7 triggers RFP-XIK tail re-localisation to the Golgi surface. This finding represents, thus far, the first example of a MyoB:myosin receptor co-localisation to a specific organelle in *A. thaliana*.

FRET-FLIM has been previously employed to detect in vivo protein-protein interactions on Golgi in plant cells^43,44^. Results in this study using FRET-FLIM, corroborated by pull down experiments, confirm DUF593 is essential for the in vivo interaction between GFP-MRF7 and RFP-XIK tail. It is important to note that while absence of DUF593 abolishes interaction, regions outside of the DUF593 region could facilitate interaction through the DUF593 region. In addition, while a 0.1 ns drop in lifetime reflects interaction between the pair, the calculated values for distance between the fluorophores (9.1 nm) is towards the upper end of the limit for FRET-FLIM detection (1-10 nm). We would therefore like to suggest that while these proteins may be interacting, the interaction may be facilitated within a complex. In addition, the drop in lifetime, whilst significant, is relatively small compared with FRET-FLIM measurement of other interacting Golgi proteins. This is likely due to the nature of the interaction and the conformational change within the resulting complex; we speculate that static interaction with no significant conformational changes may result in a larger drop in lifetime whilst interactions which result in dynamic and large conformational changes may result in smaller drops in lifetime as similarly discussed for mammalian cells by Ahmed et al.^42^. Future studies are required to determine the crystal structure of the pair and how interaction of a complex, and potential additional partners, may alter the conformational change of the myosin between active and inactive states. In this respect, we would like to add a note of caution regarding the calculated distances, and to also reiterate that in our experimental system the myosin fusions are headless and so unlikely to be regulated by head-tail intramolecular interactions. These results are consistent with observations that DUF593 mediates the interaction between *At*MyoB2-GFP and myosin XI-K^25^ and between *Nt*RISAP and *Nt*MYOXIpt^29^. The conservation of DUF593 in all land plants suggests a generalised myosin binding role for DUF593-contaning proteins in plant cells^25,27–29^. Comparison of the GFP-MRF7 lifetime measurements in the presence and absence of RFP-XIK suggests that the interaction simultaneously takes place on Golgi and in the cytosol. Given that MRF7 is a predicted soluble protein and that not all of the Golgi bodies in a cell display GFP-MRF7 and RFP-XIK tail, it is tempting to speculate that the two proteins could perhaps interact in the cytosol, resulting in XI-K recruitment to the Golgi (Fig. 8a, b). It is thus far unclear how only certain Golgi bodies are selected by MRF7 and XI-K tail.

Aside from the clear effect that GFP-MRF7 produces on XI-K tail localisation, RFP-XIK exerts an effect on MRF7, increasing the Golgi localised population. Moreover, over-expression of RFP-XIK tail leads to a gradual disappearance of the typical MyoB/MRF7 puncta from the cytosol. Although the nature of the MyoB puncta is still unclear, it was previously suggested that they are of membranous nature, either vesicles or sub domains on unknown organelles^25^. Due to the disappearance of the puncta in the absence of the DUF593 domain (Fig. 2d–f) and the observation that the interaction between GFP-MRF7 and RFP-XIK tail can take place both on Golgi and in the cytosol, it is tempting to speculate that the MRF7 interaction with a non-functional myosin could impair the ability of MRF7 to cycle on and off the Golgi. The depletion of MRF7 puncta from the cytosol following XIK tail co-expression could then be a direct consequence of impaired cycling.

The finding that XI-K tail can be targeted to the Golgi in the presence of its receptor represents an important step forward in understanding how myosins localise to target organelles. The specificity of the MRF7 effect on XI-K tail localisation was tested by expressing other myosin tails in the presence and absence of the receptor. No significant effect was observed upon co-expression of MRF7 with XI-A tail: the percentage of Golgi displaying MRF7 remains consistent before and after co-expression and XI-A tail is solely cytosolic (Fig. 7c, d). These results are consistent with the observation that myosin XI-A tail is not involved in regulation of Golgi movement in tobacco leaves^16^. MRF7 triggers the relocation of XI-1 tail, although its re-localisation from the cytosol to the Golgi is less marked than that of XI-K tail (Fig. 7a, b). This result is consistent with the observation that myosin receptors can interact with multiple myosins from class XI^27^. It indicates that MRF7 can preferentially, although not exclusively, affect XI-K localisation. As a putative soluble protein, the MRF7 ability to cycle on and off the Golgi could be fundamental to regulate the nature and number of myosin molecules recruited to the organelle which could explain the heterogenous dynamics displayed within the Golgi population. For example, MRF7 triggering localisation of myosins with different mechanochemical properties could change the speed at which Golgi move^52^, and could explain how individual Golgi seem to change their movement rates over a relatively short period of time.

MyoB family appears to be functionally redundant and so recruitment of Golgi localised myosins could also be controlled via an MRF7 independent pathway. It is tempting to speculate that there may be co-regulation between these pools of Golgi resident myosins (Fig. 8c). In our experimental system, this could explain why Golgi movement increased in response to expression of GFP-ΔMRF7; myosins with higher processivity are recruited/active on the surface of Golgi driving their movement. Golgi movement in *Arabidopsis xi-k* and *xi-2* single mutants are significantly reduced^13^ indicating functional redundancy with respect to their effects on Golgi movement. It is tempting to speculate therefore that perhaps in our experimental system, the increase in movement upon GFP-ΔMRF7 expression maybe due to another myosin (perhaps XI-2) controlling movement over XI-K whose recruitment has been affected. Intriguingly, there is evidence for several motor proteins being active or localised to the same cargo simultaneously whereby they act antagonistically to control cargo movement; for example, oskar mRNA (cargo) localisation in the Drosophila embryo is controlled by competition between myosinV and kinesin-1^53^. Here, we suggest a similar mode of action whereby multiple myosins act together to control Golgi movement; unclear whether this could be competitively (as for oskar mRNA) or through changes in activity at the Golgi surface (as shown in Fig. 8c) which could possibly be modulated through environmental/local signals. Note, MyoVa and MyoVb were recently shown to collocate to the same population of neuronal vesicles with the authors proposing recruitment of inactive myosins to the cargo with subsequent activation on the vesicle surface controlled through a local signal^54^. Future studies are required to understand how MRF7, and other potential receptors regulate myosin recruitment to the Golgi surface which ultimately controls Golgi dynamics.

It is also important to consider how myosin recruitment to Golgi could impact on ER-Golgi interaction at the ER exit sites (ERES). Here, the secretory unit model posits that the Golgi are attached to, and move with, the ERES^55^. Here, myosin recruitment to Golgi could drive Golgi movement which in turn may affect the movement of the ER. While there are correlations between the movement of the 2 organelles, the hypothesis of whether movement is coordinated by independent organelle specific myosins, or is interdependent with myosin attachment to one organelle in turn driving the movement of the other attached organelle is unclear. In addition, it is interesting to note that the localisation pattern of MRF7 as a ‘halo’ surrounding the Golgi membrane marker is similar to the localisation pattern displayed by Golgi matrix proteins, Golgins. CASP, a Golgin, affects Golgi tethering to the ER which when affected alters the speed of Golgi^56^. It is therefore interesting to speculate that perhaps MRF7s effect on Golgi movement may be through controlling Golgi tethering to the ER. Future studies are therefore required to dissect the interactions at the ER-Golgi interface to understand the regulation of MRF7 recruitment and its role in controlling Golgi dynamics. Considering complementary approaches identified interaction between MRF7 and XI-K, FRET distances indicate they are towards the upper limit approaching 10 nm which could be indicative of protein complex formation with additional interactors.

Taken altogether, these results suggest that MRF7 could act as a “universal” adaptor which mediates the recruitment to the Golgi surface of myosins involved in the regulation of Golgi movement. Future studies are required to determine whether MRF7 directly interacts with a range of myosins, or whether XI-K may heterodimerise with other myosins to regulate their MRF7 dependent Golgi recruitment. This is possible considering XI-1 was not isolated in the pull downs from transgenic GFP-MRF7 *Arabidopsis*, yet MRF7 appears to control XI-1 recruitment to Golgi in transient expression studies. Interestingly, data from expression of XI-1 truncations indicated that class XI myosin dimerisation may be required for organelle targeting^57^.

This study shows that myosins from *A. thaliana* class XI can be targeted to specific organelles upon co-expression with their adaptor/receptor counterpart on the organelle membrane. Although passive, indirect, movement of organelles in the wake of cytoplasmic streams can, to an extent, play a role in organelle movement^26^, this study demonstrates that a degree of specificity is achievable for direct recruitment of myosins to organelles. These results therefore favour the traditional over the cytoplasmic streaming-based model for MyoB dependent control over organelle movement in *Arabidopsis*.

This important step forward indicates that we will be able to specifically perturb the molecular components involved in myosin recruitment to specific organelle types with a view to specifically affecting their movement. Our current understanding of the functional role of organelle movement is largely based on experimental changes in the global organelle population rather than specific types of the organelle. Considering changes in global organelle movement has significant roles in cell growth and plant development, it is important that we begin to decipher the specific roles played through specific movement and interactions between organelles. Future studies to further define how MRF7 is targeted to the Golgi, the control mechanisms behind MRF7-myosin interaction and regulation of recruitment to the Golgi will hopefully enable the community to generate molecular tools which specifically alter Golgi movement, without perturbing the movement of other organelles, with a view to ascribing a functional role to Golgi movement.

## Methods

### Plant material and growth conditions

All wild-type and transgenic *A. thaliana* plants used in this study are in Columbia-0 (Col-0) background. They were germinated and grown in a growth chamber (Sanyo) at 20 °C on a 16 h light, 8 h dark cycle.

All *Nicotiana tabacum* plants used in this study are wild-type *cv*. Petit Havana SR1. Plants were germinated and grown in a controlled environment chamber at 20 °C on a 16 h light, 8 h dark cycle. Following infiltration, they were placed in a growth chamber (Sanyo) at 21 °C on a 14 h light, 10 h dark cycle and 70% humidity.

Generation of *A. thaliana* plants stably overexpressing genes of interest was achieved by floral dipping of Col-0 plants^58^. Transgenic seedlings were identified based on sowing seeds on 15 µg/mL phosphinotricin and screening established seedlings for GFP fluorescence. *A. thaliana* plants stably overexpressing GFP-MRF7 or GFP-ΔMRF7 were crossed with lines overexpressing the Golgi marker ST-mRFP (Chris Hawes group, Oxford Brookes University).

### Cloning and expression of fluorescently tagged proteins

MRF7 genomic sequence was amplified from gDNA of 4 week-old *A. thaliana* plants. MRF7 truncations were generated by PCR amplification from the full-length sequence. PCR products thus obtained were first cloned into pDONR207 and subsequently in the Gateway® destination vectors pB7FWG2 or pB7WGF2 to allow for C- or N-terminal GFP tagging respectively. All fluorescent fusions are driven by the CaMV35S promoter. Destination vectors were then transformed into *Agrobacterium tumefaciens* for expression in plants. Transient expression in 5 week-old tobacco leaf epidermis was achieved by *A. tumefaciens*-mediated infiltration^59^. Unless otherwise stated, Golgi and actin markers were infiltrated at a final OD_600_ = 0.04, other constructs were infiltrated at a final OD_600_ = 0.1. Bacteria suspensions at the appropriate concentrations were infiltrated with a needle-less syringe through the abaxial surface of tobacco leaves. Imaging was performed on a confocal microscope 2 days post-infiltration. Each independent infiltration was carried out using at least 2 tobacco plants per infiltration.

### Protein extraction and co-immunoprecipitation

Wild-type Col-0 *A. thaliana* seedlings and seedlings over-expressing GFP-MRF7 and GFP-ΔMRF7 were germinated and grown on ½ MS agar plates for two weeks. Whole seedlings were then harvested, flash frozen and ground in liquid nitrogen and the total protein content was extracted. Protein concentration was quantified by Bradford quantification assay^60^, according to the Bradford reagent manufacturer’s protocol. Co-immunoprecipitation was carried out using the GFP-Trap^®^_A beads technology (Chromotek) and samples thus obtained were analysed through LC–MS (University of Bristol).

For western blot analysis, 10 µg of total protein extract and 20 µl of beads-purified samples were initially separated on a 4–15% acrylamide gel by SDS-PAGE, transferred onto a PVDF membrane by standard blotting and detected with an α-GFP antibody (Abcam ab290, 1:2500).

### LC–MS

Samples were separated using SDS-PAGE, with electrophoresis carried out until the dye front had moved approximately 1 cm into the separating gel (in these circumstances, SDS-PAGE was effectively used as a clean-up step, rather than as a way of separating out individual protein bands). The 1 cm gel lane containing the entire GFP-Trap sample was then excised, digested with trypsin and the resulting peptides analysed using LC–MS as described previously^61^. The raw data files were processed and quantified using Proteome Discoverer software v1.4 (Thermo Scientific) and searched against the UniProt *A. thaliana* database (77374 sequences; downloaded May 2018) using the SEQUEST algorithm. Peptide precursor mass tolerance was set at 10ppm, and MS/MS tolerance was set at 0.8 Da. Search criteria included carbamidomethylation of cysteine (+57.0214) as a fixed modification and oxidation of methionine (+15.9949) as a variable modification. Searches were performed with full tryptic digestion and a maximum of 1 missed cleavage was allowed. The reverse database search option was enabled, and all peptide data was filtered to satisfy false discovery rate (FDR) of 5%. Data thus obtained from wild-type, GFP-MRF7 and GFP- ΔMRF7 samples were pairwise analysed as follows: wild-type/GFP-MRF7 and wild-type/GFP-ΔMRF7. All hits detected in the wild-type, but not in the transgenics samples were excluded. Of the ones detected only in the transgenics or in both the transgenic and the wild-type, only those with a #PSM value higher than 5 were included. Hits thus filtered were then compared between the two pairwise analysis to highlight potential binding partners of GFP-MRF7 and GFP-ΔMRF7.

### Latrunculin B treatment for actin depolymerisation

The actin polymerisation inhibitor Latrunculin B (LatB, Sigma Aldrich) was stored at −20 °C as a 10 mM stock solution in DMSO. The working solution was made up fresh by diluting the stock in distilled water to a final concentration of 25 µM. A 0.5 × 0.5 cm^2^ square of leaf was excised from the plant and divided into 4 smaller squares which were left floating for 1 h on 500 µL of LatB working solution. Controls were performed by following the same procedure but leaving the leaf squares floating on 500 µL of a 0.25% (v/v) DMSO solution.

### Confocal microscopy

Leaf pieces of about 5 mm^2^ were excised two days post-infiltration and expression monitored by laser scanning confocal microscopy using a Zeiss LSM 510 Meta or a Leica SP5-AOBS. On the Leica SP5, expression was analysed with a 63x HCX PL APO lambda blue (NA 1.4) oil immersion objective. CFP, GFP and mRFP were excited at 405 nm, 488 nm and 561 nm respectively. Emissions were detected with 460-480 nm, 500-515 nm and 585-595 nm filters for CFP, GFP and mRFP respectively. All images were captured using bidirectional line switching with 512 × 512 pixel resolution, using a digital zoom of 6x and 8x line average. Movies were generated using bidirectional line switching, with 512 × 256 pixel resolution, at a digital zoom of 6x, with 1x or 2x line average for 100 frames. Scan rate was 1.8 fs^−1^. On the Zeiss LSM 510 Meta, expression was analysed with a 63x oil immersion objective. CFP, GFP and mRFP were excited at 405 nm, 488 nm and 543 nm respectively. Emissions were detected using 470-500 (CFP), 505-530 (GFP) and 560–615 (mRFP) band pass filters. All images captured using bidirectional line switching at 512 × 512 pixel resolution, using a 2.8x digital zoom. Movies were taken at 256 × 256 pixel resolution, with 1x line average and 150 × 125 pixel ROI for 100 frames. Scan rate was 5 fs^−1^. Only the cortical region of leaf epidermal cells was imaged. Unless otherwise stated, no treatment was carried out before imaging.

### FRET-FLIM

*N. tabacum* leaf epidermis samples were excised and treated with Latrunculin B and FLIM data were collected using a one-photon time correlated single-photon counting (TCSPC) PC module (SPC150, Becker and Hickl GmbH) coupled to a Nikon confocal system (Central Laser Facility, Research Complex at Harwell). The FLIM platform was assembled and calibrated by the Central Laser Facility lead, Prof Stan Botchway prior to use. The confocal and lifetime set up was assembled as follows. The system is equipped with a SuperK EXTREME NKT-SC 470-2000 nm supercontinuum laser (NKT Photonics) which generates at 80 MHz repetition rate with 70 ps pulse width. The desired wavelengths were selected using a SuperK SELECT 29 multi-line tunable filter (NKT photonics). Prior to FLIM, confocal imaging of GFP, RFP and transmitted light was carried out on a Nikon Ti-E microscope with a Nikon D-eclipse C1 scan head (EZ-C1 v.3.91 software, Nikon). GFP and RFP were excited at wavelengths of 488 nm and 561 nm, respectively and imaged using a Nikon 60x VC (NA 1.20) with water immersion objective, at a field zoom of 50 µm. Fluorescence emissions of GFP and RFP were selected with a Nikon 520 ± 35 and a Comar 633IU filters, respectively. Following confocal imaging, to ensure the presence and expression of the chromophores, GFP lifetime was measured with a PMH100 detector and raw FLIM data were generated by the TCSPC software v.9.77 (Becker and Hickl, GmbH). All data presented here were collected in the “Scan Sync In” mode, with a 128 × 128 resolution and a pixel dwell time of 7.92 µs. Raw FLIM images were analysed with the SPCImage software v.6.9 (Becker and Hickl, GmbH); an incomplete multiexponential fit model with a laser repetition time value of 12.5 ns was used for the decay curve fitting. Lifetime values with χ^2^ between 0.8 and 1.3 were taken into account as a good explanatory decay fit. GFP lifetime was measured both on individual Golgi bodies and in the cell cytosol. GFP lifetimes from donor alone (GFP fusion) and samples co-expressing donor (GFP fusion) and acceptor (RFP fusion) were measured. Samples sizes are denoted in the figure legends and Supplementary Table 1. Statistical analysis of the variation in lifetimes was performed on the large datasets. The FLIM system is robust to measure changes in lifetime down to 40 ps, a 100 ps drop in lifetime therefore reflects a *bona fide* protein-protein interaction.

The energy transfer efficiencies (*E*_FRET_) and estimated molecular distances (*r*) between donor and acceptor were calculated as follows.

1 $${E}_{{{{\mathrm{FRET}}}}}=1-\frac{{\tau }_{DA}}{{\tau }_{D}}$$

2 $$r={R}_{0}.\root 6 \of{\frac{1}{{E}_{{{{\mathrm{FRET}}}}}}-1}$$

*R*_0_ represents the Fӧrster radius, the distance at which 50% of FRET occurs. *R*_0_ will vary based on the donor and acceptor molecules of choice; for the FRET pairs used in this study, *R*_0_ = 5.4 nm^62^.

### Accession numbers

Sequence data of genes used this work can be obtain on GenBank/EMBL databases using the following accession numbers: At2g24140 (MRF7), At5g20490 (XI-K), At1g04600 (XI-A), At1g17580 (XI-1), At5g66030 (GRIP), At2g24765 (ARL1). For accession numbers of ST Golgi marker^63^, and LifeAct actin marker^64^ refer to the original papers.

### Quantification

All quantifications were carried out from movies or still confocal images acquired as described in the “Confocal microscopy” section. Golgi localisation of GFP-tagged fusions was quantified from still images. The total number of Golgi bodies (labelled by the Golgi marker ST) in an imaged area were counted. Of those, Golgi bodies where the GFP-tagged fusion surrounded the ST marker like a halo were considered as showing surface localisation. The percentage of Golgi showing surface localisation, over the total number of Golgi bodies, was calculated for each image.

Quantification of Golgi and puncta dynamics was carried out from movies using either Imaris (BitPlane) or the ImageJ (NIH) plugin TrackMate. The following parameters were quantified: track speed (µm/s), calculated as the entire length of the track over time; track straightness index (A.U.), calculated as displacement rate divided by track speed, is an indicator of movement directionality and therefore of track straightness (i.e., if displacement rate and speed are the same the track straightness index is 1 and indicates the object has moved in a straight line).

All kymographs presented in Supplementary Figs. 2 and 3 were generated from time series of Golgi bodies or GFP-MRF7 puncta using the MultiKymograph tool from ImageJ.

### Statistics and reproducibility

Graphical representations and statistical data analysis were generated with GraphPad Prism 8. Format of data representation and statistical tests were chosen on a case-to-case basis, depending on the nature of the data collected. For statistical analysis, data were collected from at least three independent experiments. Where possible, an equal number of images and movies were collected and analysed for both controls and the experimental condition of interest. Normally distributed data was statistically analysed with a pairwise Student’s *t*-test. Non-normally distributed data was statistically analysed with a Mann–Whitney test. Specifications on the choice of tests and exact *p*-values are provided within each section and figure caption.

### Reporting summary

Further information on research design is available in the Nature Research Reporting Summary linked to this article.

## Supplementary information

Supplementary information

Description of additional supplementary material

Supplementary Data 1

Supplementary Data 2

Supplementary Data 3

Supplementary Data 4

Supplementary Data 5

Reporting Summary

## Data Availability

All the raw data used to generate the figures is available within the Supplementary Data files. The mass spectrometry proteomics data have been deposited to the ProteomeXchange Consortium (http://proteomecentral.proteomexchange.org) via the PRIDE partner repository^65^ with the dataset identifier PXD027155. Other data is available upon request.
