# Supplementary material for: Arabidopsis thaliana myosin XIK is recruited to the Golgi through interaction with a MyoB receptor
Source: Commun Biol. 2021 Oct 13;4:1182. doi: 10.1038/s42003-021-02700-2 (PMC8514473; doi:10.1038/s42003-021-02700-2)
Supplement: Supplementary file 1 — Supplementary information [file 42003_2021_2700_MOESM1_ESM.pdf]

Perico et al – Supplementary information

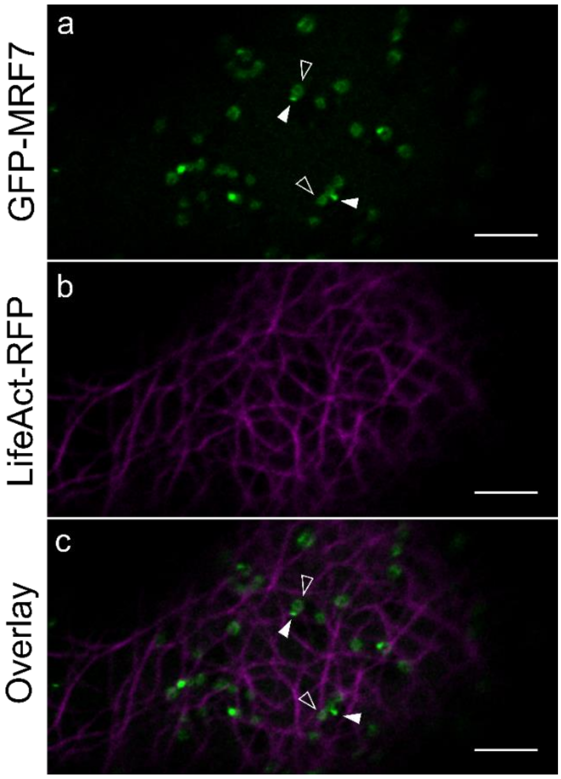

Supplementary Fig 1. Co-existence of GFP-MRF7 puncta and GFP-MRF7 Golgi-like structures on the actin cytoskeleton. **(a, c)** Representative image showing the coexistence of GFP-MRF7 puncta (filled arrowheads) and Golgi-like structures (empty arrowheads) along the actin cytoskeleton. **(b, c)** Actin is labelled by the LifeAct-RFP marker. Imaging was carried out two days post-infiltration on transiently transfected tobacco leaf epidermal cells. All scalebars = 5 µm.

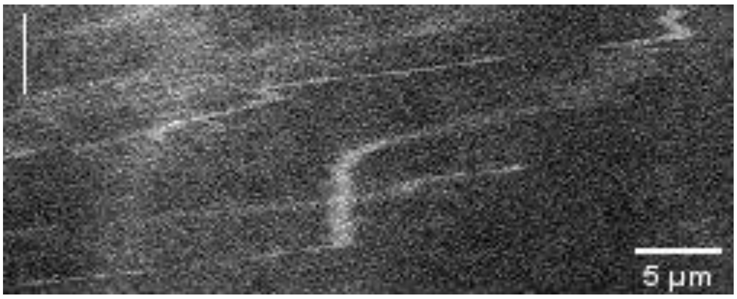

Supplementary Fig 2: Representative kymograph of time series following GFP-MRF7 puncta movement in tobacco leaf epidermal cells. Kymograph generated from time lapse series in cells expressing GFP-MRF7 only. Trajectories indicate the movement of GFP-MRF7 puncta over time. Time is represented on the y-axis (bar = 5s).

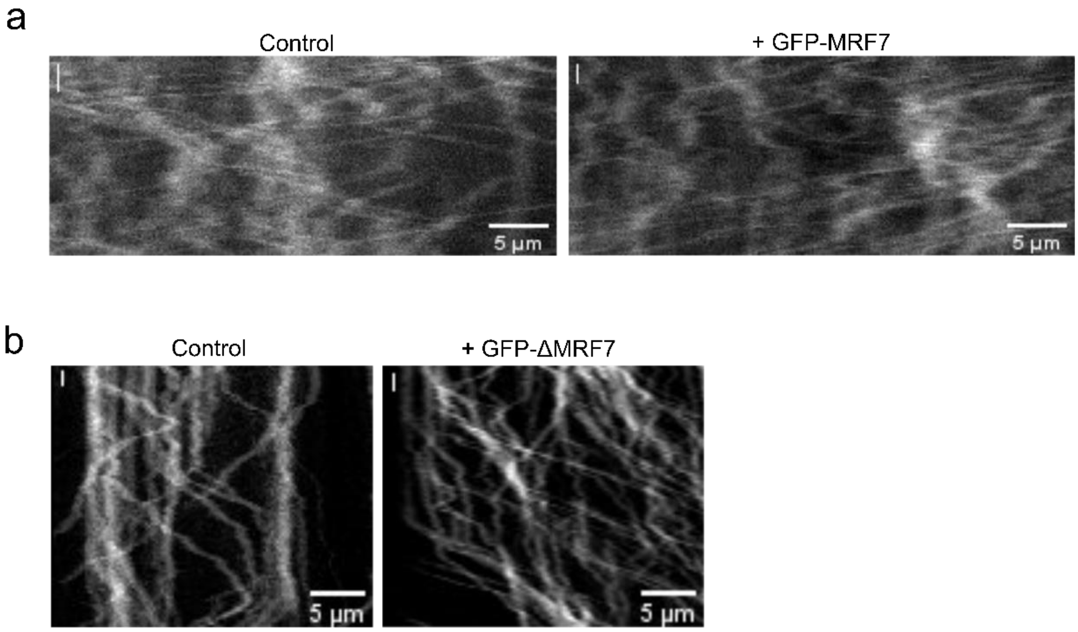

Supplementary Fig 3: Representative kymograph of times series following Golgi movement in the presence and absence of either GFP-MRF7 or GFP-ΔMRF7 in tobacco leaf epidermal cells. a) Representative kymograph of time series of Golgi movement in the presence and absence (control) of GFP-MRF7. Time is represented on the y-axis (bar = 5s). **b)** Representative kymograph of a time series of Golgi movement in the presence and absence (control) of GFP-ΔMRF7. Time is represented on the y-axis (bar = 1s).

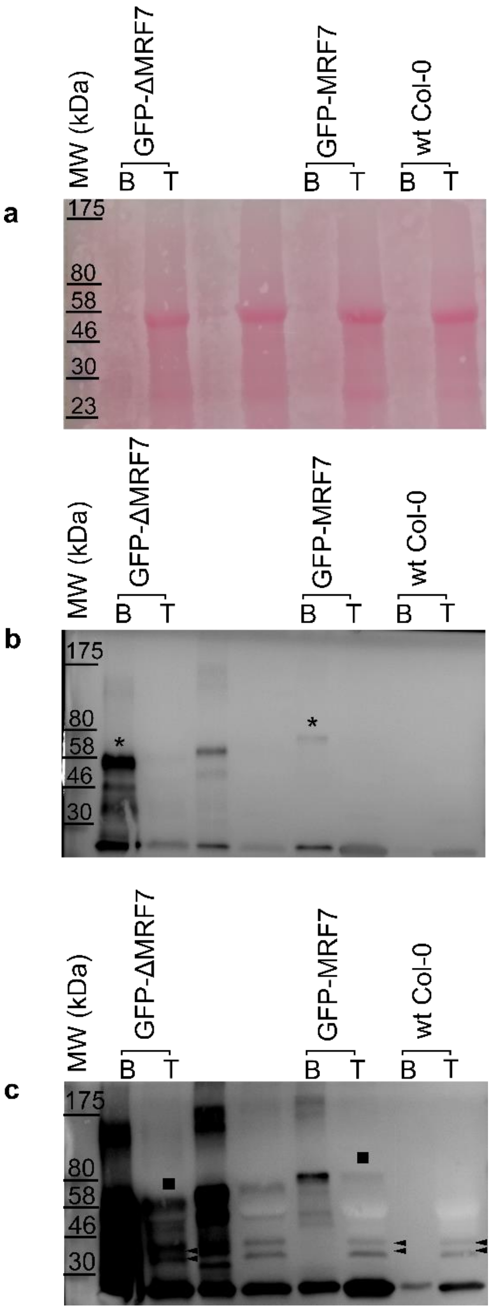

Supplementary Fig 4. Expression of MRF7 fusions in *A.thaliana* total protein extract and beads purified samples. The SDS-PAGE gel was loaded with 10 µg of total protein extract (“T”) and 20 µl of beads-purified samples (“B”) from GFP-MRF7, GFP-ΔMRF7 and wild-type Col-0 plants. **(a)** Ponceau staining of the PVDF membrane: bands corresponding to the RuBisCo large subunit are visible between 46 kDa and 58 kDa marker bands in the “T” labelled wells. **(b)** Membrane from (A) was developed with a standard ECL solution for 30s: asterisks indicate the bands corresponding to GFP-MRF7 and GFP-ΔMRF7 in the beads-purified sample (“B”). A non-specific band was detected in the lower part of the blot (<30 kDa). **(c)** Membrane was washed and developed with the SuperSignal ECL solution for 4s. Bands corresponding to the fusion proteins can now be seen also in the total protein extract wells (“T”, black squares). Arrowheads indicate non-specific bands detected by the α-GFP antibody in the total protein extract samples. Please note that the unlabelled lanes 3 and 4 should not be considered as they are not relevant to this study.

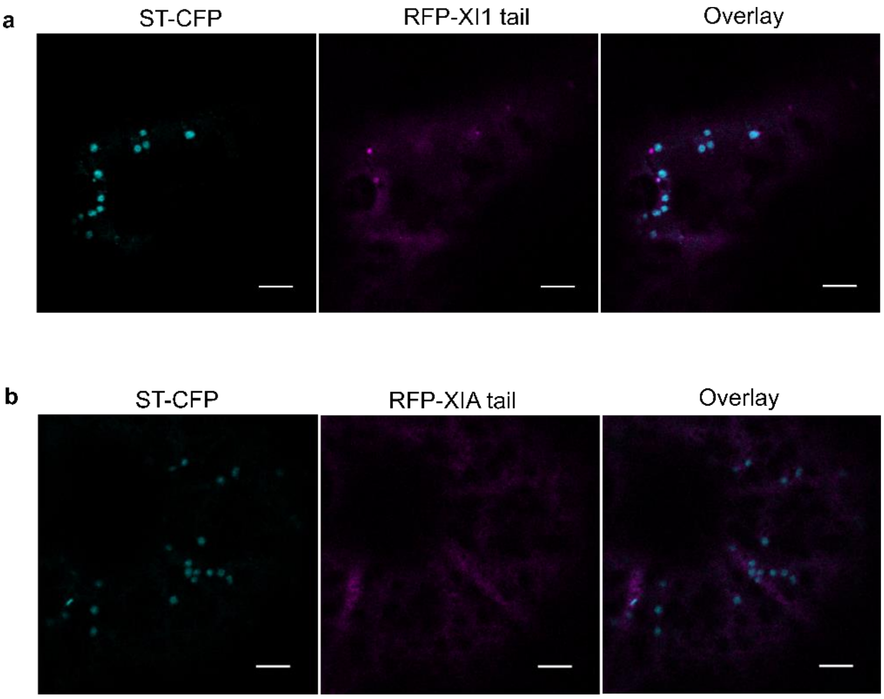

Supplementary Fig 5. Localisation of myosin XI-1 and XI-A tails in the absence of GFP-MRF7. (**a**) RFP-XI1 tail localises to the cytoplasm and occasionally, on punctate structures; no trace of the myosin is found on the Golgi, labelled by the marker ST-CFP. (**b**) RFP-XIA tail localises to the cytoplasm. As in (a), ST-CFP labels the Golgi stacks. Imaging carried out two days post infiltration. All scale bars = 5 µm.

FRET pairs	N Golgi	N cells	N experiments
GFP-MRF7	99	16	4
GFP-MRF7 + RFP-XIK tail	101	15	4
GFP-ΔMRF7	92	13	3
GFP-ΔMRF7 + RFP-XIK tail	81	12	3
ST-GFP	158	11	4
ST-GFP + ST-RFP	151	10	4
GRIP-GFP	155	13	4
GRIP-GFP + RFP-ARL1	83	12	4

Supplementary Tab 1. Sample size for FRET pairs used for lifetime measurements.

Myosin tail	Percentage of Golgi with MRF7 before tail expression	Percentage of Golgi with MRF7 after tail expression	p-value
XI-K	26 %	62 %	0.0005
XI-1	15 %	52 %	0.002
XI-A	15 %	23 %	0.1645

Supplementary Tab 2. Average percentage of Golgi displaying GFP-MRF7 in the presence and absence of XI-K, XI-1 and XI-A tails. P-values calculated with the Mann Whitney U test.

Myosin tail	Golgi with myosin tail before MRF7 expression	Golgi with myosin tail after MRF7 expression	p-value
XI-K	0 %	36 %	< 0.0001
XI-1	0 %	16 %	0.0008
XI-A	0 %	0 %	> 0.9999

Supplementary Tab 3. Average percentage of Golgi displaying myosin XI-K, XI-1 and XI-A tails in the presence and absence of GFP-MRF7. P-values calculated with the Mann Whitney U test.
