# Supplementary material for: Arabidopsis thaliana myosin XIK is recruited to the Golgi through interaction with a MyoB receptor
Source: Commun Biol. 2021 Oct 13;4:1182. doi: 10.1038/s42003-021-02700-2 (PMC8514473; doi:10.1038/s42003-021-02700-2)
Supplement: Supplementary file 2 — Description of additional supplementary material [file 42003_2021_2700_MOESM2_ESM.pdf]

Description of Additional Supplementary Files

File name: Supplementary Data 1

Description: Raw values of speed, track straightness index and diameter of the GFP-MRF7 puncta. The data were used to generate Figure 1h, i and j, respectively.

File name: Supplementary Data 2

Description: Raw values of Golgi speed and percentage of Golgi localisation per cell for GFP-MRF7, GFP-ΔMRF7 and GFP-ΔMRF7ΔC. The data were used to generate Figure 2 j-l and Figure 3g.

File name: Supplementary Data 3

Description: Summary of the filtered LCMS results from 2 independent repeats. Filtered result show either unique or enriched interactors of GFP-MRF7 or GFP-ΔMRF7 when compared to Columbia WT controls.

File name: Supplementary Data 4

Description: Raw values of the percentage of Golgi localisation per cell for myosin XIK, XI1 and XIA tails, in the presence and absence of GFP-MRF7. The data were used to generate Figure 5c and Figure 7b, d.

File name: Supplementary Data 5

Description: Raw lifetime values used to generate Figure 6.
