# Supplementary material for: Arabidopsis thaliana myosin XIK is recruited to the Golgi through interaction with a MyoB receptor
Source: Commun Biol. 2021 Oct 13;4:1182. doi: 10.1038/s42003-021-02700-2 (PMC8514473; doi:10.1038/s42003-021-02700-2)
Supplement: Supplementary file 8 — Reporting Summary [file 42003_2021_2700_MOESM8_ESM.pdf]

nature research

Corresponding author(s): Dr Imogen Sparkes

Last updated by author(s): Aug 25, 2021

Reporting Summary

Nature Research wishes to improve the reproducibility of the work that we publish. This form provides structure for consistency and transparency in reporting. For further information on Nature Research policies, see our Editorial Policies and the Editorial Policy Checklist.

Statistics

For all statistical analyses, confirm that the following items are present in the figure legend, table legend, main text, or Methods section.

n/a	Confirmed	
☐	☒	The exact sample size (*n*) for each experimental group/condition, given as a discrete number and unit of measurement
☐	☒	A statement on whether measurements were taken from distinct samples or whether the same sample was measured repeatedly
☐	☒	The statistical test(s) used AND whether they are one- or two-sided<br>*Only common tests should be described solely by name; describe more complex techniques in the Methods section.*
☒	☐	A description of all covariates tested
☒	☐	A description of any assumptions or corrections, such as tests of normality and adjustment for multiple comparisons
☐	☒	A full description of the statistical parameters including central tendency (e.g. means) or other basic estimates (e.g. regression coefficient) AND variation (e.g. standard deviation) or associated estimates of uncertainty (e.g. confidence intervals)
☐	☒	For null hypothesis testing, the test statistic (e.g. F, t, r) with confidence intervals, effect sizes, degrees of freedom and P value noted<br>*Give P values as exact values whenever suitable.*
☒	☐	For Bayesian analysis, information on the choice of priors and Markov chain Monte Carlo settings
☒	☐	For hierarchical and complex designs, identification of the appropriate level for tests and full reporting of outcomes
☒	☐	Estimates of effect sizes (e.g. Cohen's d, Pearson's r), indicating how they were calculated

Our web collection on statistics for biologists contains articles on many of the points above.

Software and code

Policy information about availability of computer code

Data collection	TCSPC software v.9.77 for FRET FLIM
Data analysis	GraphPad Prism 8, SPCImage software v.6.9

For manuscripts utilizing custom algorithms or software that are central to the research but not yet described in published literature, software must be made available to editors and reviewers. We strongly encourage code deposition in a community repository (e.g. GitHub). See the Nature Research guidelines for submitting code & software for further information.

Data

Policy information about availability of data

All manuscripts must include a data availability statement. This statement should provide the following information, where applicable:

- Accession codes, unique identifiers, or web links for publicly available datasets
- A list of figures that have associated raw data
- A description of any restrictions on data availability

All data generated or analysed in this study are included in this published article and its supplementary information files.

Field-specific reporting

Life sciences study design

All studies must disclose on these points even when the disclosure is negative.

Sample size	All analyses were carried out on data collected from at least three independent experiments. Details are provided in the manuscript on sample size.
Data exclusions	No data were excluded from this study.
Replication	Experiments were replicated at least three times.
Randomization	Randomization here was based on unbiased selection of independent cells on the microscope. Using a grid based scanning approach across the sample to select the first cell which contains the relevant fusion proteins and then moving at least 10 cells diameters to the next area.
Blinding	Where the experimentalist collected and analysed the data blinding was not possible.

Reporting for specific materials, systems and methods

We require information from authors about some types of materials, experimental systems and methods used in many studies. Here, indicate whether each material, system or method listed is relevant to your study. If you are not sure if a list item applies to your research, read the appropriate section before selecting a response.

Materials & experimental systems

n/a	Involved in the study	
☐	☒	Antibodies
☒	☐	Eukaryotic cell lines
☒	☐	Palaeontology and archaeology
☐	☒	Animals and other organisms
☒	☐	Human research participants
☒	☐	Clinical data
☒	☐	Dual use research of concern

Methods

n/a	Involved in the study	
☒	☐	ChIP-seq
☒	☐	Flow cytometry
☒	☐	MRI-based neuroimaging

Antibodies

Antibodies used	Rabbit Polyclonal IgG anti-GFP (1:2500), Abcam, ab290. Goat anti-rabbit IgG - HRP (1:5000), Abcam, ab97200
Validation	ab290 and ab97200 are two widely used commercially produced antibodies suitable for western blot. The specificity of the detection was tested on wild-type plants not expressing GFP-tagged proteins, as reported in Supplemental Fig 1.

Animals and other organisms

Policy information about studies involving animals; ARRIVE guidelines recommended for reporting animal research

Laboratory animals	No lab animals
Wild animals	No wild animals
Field-collected samples	No field collected samples
Ethics oversight	None. Plant material.

Note that full information on the approval of the study protocol must also be provided in the manuscript.
